# Exploring and validating associations between six systemic inflammatory indices and ischemic stroke in a middle-aged and old Chinese population

**DOI:** 10.1007/s40520-024-02912-6

**Published:** 2025-01-21

**Authors:** Yulu Zheng, Zheng Guo, Jingzheng Wang, Zhiyuan Wu, Xiaolin Chen, Yahong Zhu, Guangle Shan, Haifeng Hou, Xingang Li

**Affiliations:** 1 Centre for Precision Health, Edith Cowan University, Perth, WA Australia; 2 Dongping People’s Hospital, Tai’an, Shandong China; 3 Harvard T H Chan School of Public Health, Boston, MA USA; 4 Department of Orthopedic Surgery, The Second Affiliated Hospital of Chongqing Medical University, Chongqing, China; 5 School of Science, Edith Cowan University, Perth, WA Australia; 6 Department of Bioinformatics, Thrive Bioresearch, Beijing, China; 7 School of Public Health, Shandong First Medical University and Shandong Academy of Medical Sciences, Tai’an, Shandong China; 8 The Second Affiliation Hospital of Shandong First Medical University, Tai’an, Shandong China

**Keywords:** Stroke, Systemic inflammation, Middle-aged and old population

## Abstract

**Background:**

Inflammation and maladaptive immune mechanisms have been substantiated as integral components in the critical pathological processes of the injury cascade in ischemic stroke (IS). This study aimed to explore the associations between six systemic inflammatory indices and IS in a Chinese population.

**Methods:**

This was a case-control study based on the retrospective review of electronic medical records from two hospitals in Shandong Province, China. Systemic inflammatory indices, including the systemic inflammation response index (SIRI), systemic immune inflammation index (SII), pan-immune-inflammation value (PIV), neutrophil lymphocyte ratio (NLR), platelet lymphocyte ratio (PLR), and lymphocyte monocyte ratio (LMR), were calculated. Logistic regression models and classification analyses were employed to evaluate associations and discriminatory abilities.

**Results:**

In total, 9392 participants aged 40–83 years old were included in the discovery (3620 pairs of IS-present cases and healthy controls) and validation (1076 pairs of IS-present cases and IS-absent controls with IS mimics) datasets. After adjusting for potential confounding factors, IS was found to be associated with all six systemic indices in the discovery dataset, including SIRI (odd ratio [OR] 8.77, 95% confidence interval [CI] 7.48–10.33), SII (1.03, 1.01–1.04), PIV (1.01, 1.01–1.01), NLR (2.23, 2.08–2.39), PLR (1.01, 1.01–1.01), and LMR (0.77, 0.75–0.78). Notably, only LMR exhibited significant associations with IS in both discovery and validation datasets (0.88, 0.83–0.93), suggesting an independent protective role of this index. SIRI, SII, PIV, NLR, and LMR showed good discriminative ability between IS patients and healthy controls in the discovery dataset (AUCs > 0.70). However, they performed poorly in distinguishing IS patients from IS mimics in the validation dataset (AUCs < 0.60).

**Conclusion:**

This study provides valuable insights into the associations between systemic inflammatory indices and IS, offering potential implications for risk stratification. While these inflammatory indices are potential indicators for distinguishing IS from healthy conditions, additional biomarkers may be needed when differentiating IS from other chronic inflammatory conditions in clinical practice.

**Supplementary Information:**

The online version contains supplementary material available at 10.1007/s40520-024-02912-6.

## Introduction

Stroke stands as the world's second leading cause of mortality and the third largest cause of disability, with global incidence increasing by 70% over the last three decades [1]. The burden of stroke has increased higher in low- and middle-income nations compared to high-income countries [2]. A stroke can manifest when the brain loses blood supply due to the blockage of blood flow, leading to what is termed an ischemic stroke (IS), which accounts for 87% of all stroke cases. Alternatively, a stroke can result from a sudden bleeding blood vessel in the brain, known as a hemorrhagic stroke, accounting for the remaining 13% [3]. IS occurs when blood vessel occlusion triggers an ischemic cascade, ultimately resulting in tissue infarction [4].

In the pathophysiological processes of IS, inflammation and maladaptive immune mechanisms (involving the innate and adaptive immune systems) are confirmed to be involved in the most important pathological mechanisms in this injury cascade [5]. In ischemic brain parenchyma and blocked and hyperperfused arteries, the immune response initiates locally [6]. Meanwhile, inflammatory mediators produced in situ spread throughout the entire organism [7]. The first effect of this spillover is a systemic inflammatory response, which is then followed by immunosuppression meant to reduce the potentially hazardous proinflammatory environment [8]. As such, pathobiological procession and outcome are significantly influenced by the immune system's reactions to the initial ischemia [9].

Advancements in the field of innovative composite indices have resulted in the creation of six systemic inflammatory indices: the Systemic Inflammation Response Index (SIRI), Systemic Immune Inflammation Index (SII), Pan-Immune-Inflammation Value (PIV), Neutrophil Lymphocyte Ratio (NLR), Platelet Lymphocyte Ratio (PLR), and Lymphocyte Monocyte Ratio (LMR), These indices represent a sophisticated integration of platelets with three distinct white blood cell (WBC) subsets, namely neutrophils, monocytes, and lymphocytes. Previous studies have established the correlations between these indicators and non-communicable diseases (NCDs) such as cognitive decline [10], coronary artery disease [11], hyperlipidemia [12], hepatocellular carcinoma [13], and gastric cancer [14, 15]. Additionally, a few studies have investigated the associations between one or two of those six systemic inflammatory indices (primarily SIRI and SII) and stroke [16, 17]. These studies have provided insights into two indices' roles in stroke severity and stroke outcomes. Evidence suggests that chronic low-grade inflammation contributes to vascular endothelial injury, oxidative stress, and thrombosis, potentially serving as an underlying cause of mortality [18].

However, while previous studies have focused on SIRI and SII in IS prognosis, the effect of multiple inflammatory indices, especially in differentiating IS from stroke mimics, remains understudied. Our study seeks to address this gap by building upon the existing literature, systematically investigating the associations between six systemic inflammatory indices and IS within a case-controlled design. Understanding the nuanced relationships between these indices and IS is crucial for unraveling their potential as sensitive biomarkers for IS. This, in turn, can contribute to a more nuanced risk stratification and personalized management of individuals at risk of IS.

## Methods

### Study design and eligibility criteria

A retrospective review of the electronic medical records (EMRs) from two hospitals in Shandong Province, China, was conducted to investigate associations between systemic inflammatory indices and IS. A 1:1 age- and sex-matched case–control study assessed these associations compared to healthy controls, followed by a case–control study validating these associations against IS-absent controls with IS mimics, such as other chronic diseases or disorders. Approval was obtained from the Clinical Ethics Review Committees of two hospitals (No. 2020–066 and No. DPH-06102021, respectively), with a waiver for informed consent owing to minimal risk. This study adhered to the Strengthening the Reporting of Observational Studies in Epidemiology (STROBE) reporting guideline (Supplementary Material).

### Data collection

The investigation data were derived from the Second Affiliated Hospital of Shandong First Medical University (SAH-SFMU) from January 2015 to December 2019. Demographic information, routine hematological, and blood biochemistry tests were extracted retrospectively. The validation dataset was from Dongping People’s Hospital (DPH) between December 2019 to October 2021. It included demographic and clinical variables of IS cases and IS-absent controls with various diseases or disorders. All lab-based variables were collected during routine outpatient visits prior to the diagnosis of ischemic stroke.

### Inclusion and exclusion criteria

The Chinese Han adults aged ≥ 40 years old and without a history of a diagnosis of IS were included in this research. In both discovery and validation datasets, the IS (ICD 9: 433–434 and 436; ICD10: I63.9) was diagnosed as a sudden symptom of neurological deficit (i.e., sudden weakness, numbness, lessened control of one side of the body, sudden dimness, loss of vision in one or both eyes, loss of speech, dizziness, unsteadiness or sudden fall, and difficulty in wallowing), followed by the diagnosis of computed tomography (CT) and/or magnetic resonance imaging (MRI). The healthy controls in discovery dataset were acquired from the Regular Physical Examination Centre of SAH-SFMU. IS-absent controls in the validation dataset included the adults who had presented IS mimics-related diseases or disorders (such as type 2 diabetes, cardiovascular diseases, cancer, headache, dizziness, and limb numbness).

Exclusions comprised individuals in pregnancy or lactation, severe mental disorders, or other serious physical illnesses and injuries [19]. Participants with missing values on key parameters (i.e., age, sex, and routine hematological or blood biochemistry results) were also excluded.

### Definitions of six systemic inflammatory indices

Six systemic inflammatory indices (SIRI [20], SII [13], PIV [21], NLR [22], PLR [14], and LMR [23]) were calculated based on the values of neutrophil count, monocyte count, lymphocyte count, and platelet count (presented as × 10^9^ cells/L). The calculations were conducted as follows:

1 $$SIRI=\frac{neutrophil \, count \times monocyte \, count}{lymphocyte \, count}$$

2 $$SII=\frac{neutrophil \, count \times platelet \, count}{lymphocyte \, count}$$

3 $$PIV=\frac{neutrophil \, count \times monocyte \, count\times platelet \, count}{monocyte \, count}$$

4 $$NLR=\frac{neutrophil \, count}{lymphocyte \, count}$$

5 $$PLR=\frac{platelet \, count}{lymphocyte \, count}$$

6 $$LMR=\frac{lymphocyte \, count}{monocyte \, count}$$

### Statistical analysis

Age was presented as mean (standard deviation, [SD]), sex as count (%), and other continuous variables as median (interquartile range, [IQR]). As the distributions of six systemic inflammatory indices were skewed, in addition to comparing those indices with original values between IS-present and controlled groups, log-transformed systemic inflammatory indices were also calculated and compared. The main outcome was the associations of six systemic inflammatory indices with IS.

Wilcoxon signed-rank tests compared variables between IS-present and control groups. Logistic regression models assessed associations between inflammatory indices and IS, adjusting for relevant variables: *Model 1*: unadjusted model; *Model 2*: adjusted glucose and lipids profile (total cholesterol [TC], triglycerides [TG], low-density lipoprotein [LDL] cholesterol, high-density lipoprotein [HDL] cholesterol); *Model 3*: further adjusted hemoglobin (Hgb), uric acid (UA), total protein (TP), and calculated globulin (CG) based on Model 2. Classification models evaluated index performance in discriminating IS patients from controls using receiver operating characteristic (ROC) analysis. Sensitivity analyses were conducted, stratifying by age (aged 40–64 years old *vs* aged ≥ 65 years old) and sex (male *vs* female). In sensitivity analyses, ORs with corresponding 95% CIs were measured by logistic regression model with adjustments of glucose, TC, TG, LDL, HDL, Hgb, UA, TP, and CG.

All analyses were performed using R (version 4.0.5), with a false discovery rate (FDR) correction and significance set at *P* < 0.05 (2-tailed).

## Results

### Characteristics of the study population

**Fig. 1:**
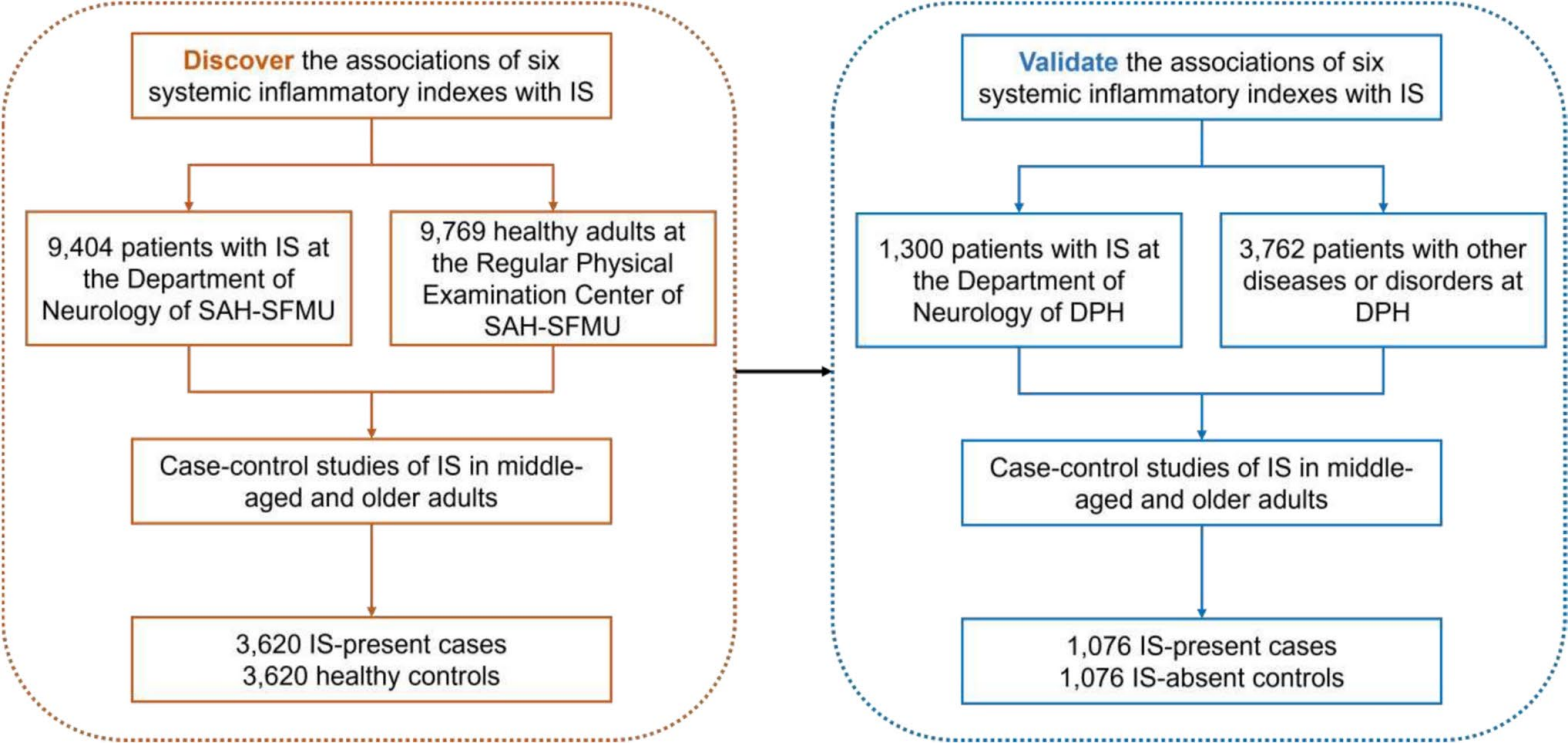
Flowchart of the study. *IS* ischemic stroke, *SAH-SFMU* the Second Affiliated Hospital of Shandong First Medical University, *DPH* Dongping People’s Hospital

After applying exclusion criteria, a total of 7240 and 2152 middle-aged and older adults from the two respective datasets were included in the final analysis (Fig. 1). In the discovery dataset, comprising 3620 IS-present cases and 3620 healthy controls (60.6% were men), significant differences in hematological and biological results were observed between IS-present and healthy control groups (*P* < 0.001). The validation dataset included 1076 IS-present cases and 1076 IS-absent controls (58.6% men), with no statistical difference in TG between IS-present and IS-absent groups. Further details are presented in Table 1.

**Table 1:** Characteristics of the study population

Variables^a^	Discovery dataset (SAH-SFMU)		Validation dataset (DPH)	
	IS-present cases	Healthy controls	IS-present cases	IS-absent controls
N	3620	3620	1076	1076
Age, mean (SD)	59.09 (8.83)	60.63 (8.91)^b^	64.54 (8.66)	65.41 (8.78) ^d^
Sex, count (%)				
Female	1427 (39.4)	1427 (39.4)	445 (41.4)	445 (41.4)
Male	2193 (60.6)	2193 (60.6)	631 (58.6)	631 (58.6)
Neutrophil count (10^9/L)	3.07 [2.51, 3.78]	4.08 [3.20, 5.26]^b^	3.99 [2.92, 3.99]	3.99 [3.26, 4.31]^b^
Monocyte count (10^9/L)	0.27 [0.22, 0.34]	0.36 [0.28, 0.48]^b^	0.44 [0.35, 0.46]	0.44 [0.38, 0.48]^c^
Lymphocyte count (10^9/L)	1.83 [1.52, 2.20]	1.77 [1.38, 2.18]^b^	1.84 [1.48, 1.93]	1.84 [1.41, 1.84]^d^
Platelet count (10^9/L)	203.00 [172.00, 240.00]	220.00 [185.00, 259.00]^b^	233.11 [205.00, 250.00]	233.11 [191.75, 235.00]^b^
Glucose (mmol/L)	5.57 [5.16, 6.25]	5.38 [4.79, 6.41]^b^	6.16 [5.52, 6.16]	5.98 [5.07, 6.17]^c^
TC (mmol/L)	5.35 [4.69, 6.03]	4.68 [4.01, 5.43]^b^	4.93 [4.93, 4.97]	4.93 [4.04, 4.93]^b^
TG (mmol/L)	1.34 [0.98, 1.91]	1.22 [0.91, 1.74]^b^	1.33 [1.05, 1.33]	1.33 [0.95, 1.38]
LDL.C (mmol/L)	2.95 [2.49, 3.44]	2.64 [2.14, 3.16]^b^	3.40 [3.39, 3.42]	3.40 [2.67, 3.50]^b^
HDL.C (mmol/L)	1.39 [1.19, 1.62]	1.21 [1.03, 1.44]^b^	1.27 [1.27, 1.29]	1.27 [1.02, 1.27]^b^
Hemoglobin (g/L)	146.00 [137.00, 156.00]	138.00 [128.00, 148.00]^b^	133.82 [131.00, 144.00]	133.82 [126.00, 137.00]^b^
Uric acid (μmol/L)	331.00 [279.00, 386.00]	302.00 [249.00, 361.00]^b^	273.86 [264.00, 273.86]	273.86 [246.00, 306.25]^c^
Total protein (g/L)	70.50 [68.00, 73.10]	66.40 [62.30, 70.80]^b^	69.13 [69.13, 70.20]	69.00 [63.90, 69.13]^b^
Calculated globulin (g/L)	27.30 [25.10, 29.60]	25.40 [22.60, 28.20]^b^	29.47 [29.40, 29.60]	29.47 [26.60, 29.90]^b^
SIRI	0.45 [0.32, 0.65]	0.83 [0.53, 1.34]^b^	0.95 [0.59, 1.02]	0.95 [0.78, 1.22]^b^
SII	339.84 [254.47, 449.67]	493.67 [349.29, 738.92]^b^	504.69 [349.62, 553.95]	504.69 [403.72, 598.80]^c^
PIV	90.82 [59.85, 137.43]	181.46 [110.73, 306.91]^b^	221.83 [123.49, 240.60]	221.83 [164.01, 273.34]^b^
NLR	1.66 [1.32, 2.10]	2.23 [1.68, 3.20]^b^	2.17 [1.61, 2.44]	2.17 [1.94, 2.71]^b^
PLR	110.88 [90.33, 136.75]	124.26 [96.88, 160.52]^b^	126.50 [110.35, 154.38]	126.50 [117.01, 150.80]
LMR	6.70 [5.28, 8.65]	4.83 [3.57, 6.56]^b^	4.19 [3.53, 5.11]	4.19 [3.31, 4.35]^b^

*IS* ischemic stroke; *SAH-SFMU* the Second Affiliated Hospital of Shandong First Medical University; *DPH* Dongping People’s Hospital; *TC* total cholesterol; *TG* triglyceride; *LDL.C* low-density lipoprotein cholesterol; *HDL.C* high-density lipoprotein cholesterol; *SIRI* systemic inflammation response index; *SII* systemic immune-inflammation index; *PIV* pan-immune-inflammation value; *NLR* neutrophil lymphocyte ratio; *PLR* platelet lymphocyte ratio; *LMR* lymphocyte monocyte ratio

^a^Values are median [IQR] unless specified otherwise. Differences of variables between IS-present and control groups were examined by paired t-tests (age), Wilcoxon signed-rank tests (results of routine hematological and blood biochemistry tests, six systemic inflammatory indices), and chi-squared test (sex)

^b^*P* < .001

^c^*P* < .01

^d^*P* < .05

**Fig. 2:**
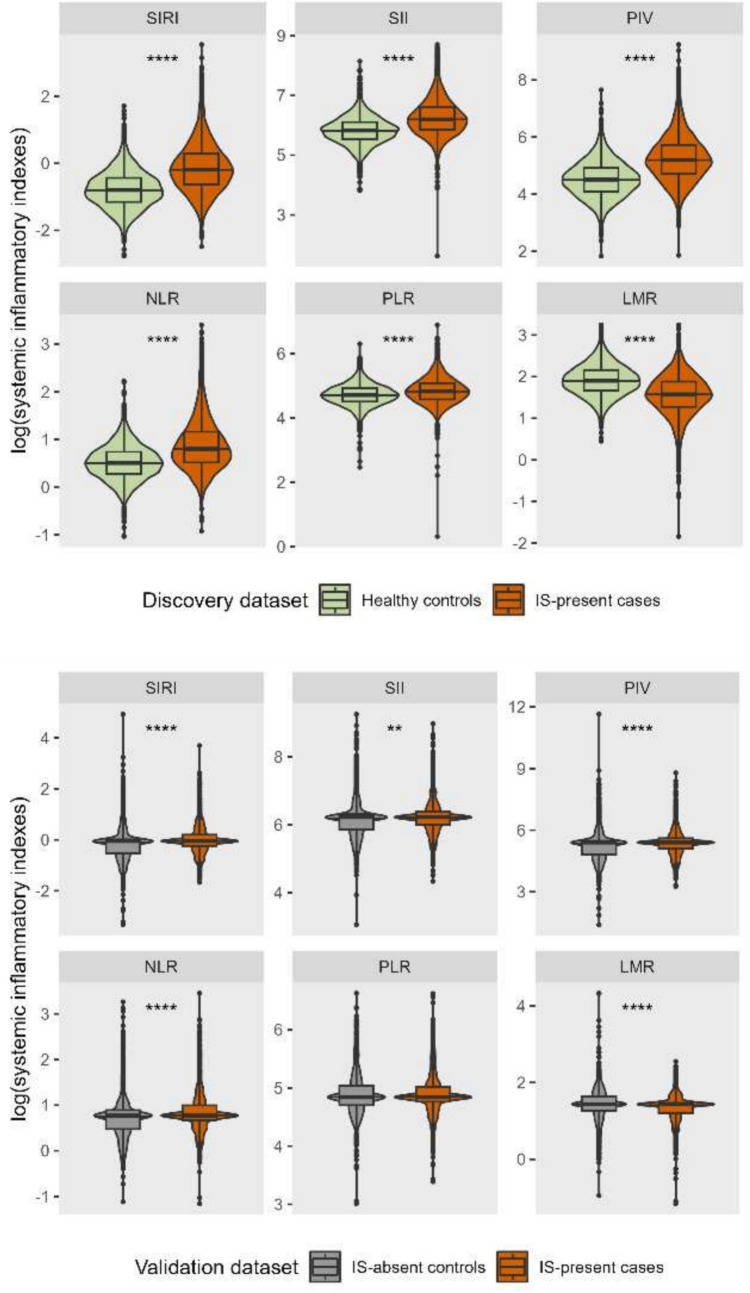
Comparisons of six log-transformed systemic inflammatory indices between case and control groups. **P < 0.01 ****P < 0.0001. *IS* ischemic stroke, *SIRI* systemic inflammation response index, *SII* systemic immune-inflammation index, *PIV* pan-immune-inflammation value, *NLR* neutrophil lymphocyte ratio, *PLR* platelet lymphocyte ratio, *LMR* lymphocyte monocyte ratio

Violin plots showed that all six log-transformed systemic inflammatory indices (SIRI, SII, PIV, NLR, PLR, and LMR) were significantly different between IS-present and healthy control groups in discovery dataset. Five systemic inflammatory indices (SIRI, SII, PIV, NLR, and LMR) showed significant differences between IS-present and IS-absent groups in validation dataset, while only PLR did not show a significant difference when comparing IS-present with IS-absent groups (Fig. 2).

### Associations of six systemic inflammatory indices and ischemic stroke

Compared to healthy controls, a 1-unit increase in SIRI was associated with 777% increase in the odds of IS (OR 8.77, 95% CI 7.48, 10.33) after adjusting for various factors, including glucose, TC, TG, LDL, HDL, Hgb, UA, TP, and CG. However, no such association was observed in validation dataset when comparing IS to IS-absent controls (OR 0.99, 95% CI 0.95, 1.02). Similar patterns were observed for SII, PIV, NLR, and PLR, showing statistically significant associations with IS-present cases compared to healthy controls in discovery dataset but not with IS-present case compared to IS-absent controls in validation dataset. LMR was negatively associated with IS in discovery dataset, with a 1-unit increase in LMR indicating a 12–23% decrease in the odds of IS (OR 0.77, 95% CI 0.75, 0.78). This negative association was supported by the validation dataset, with an OR of 0.88 (95% CI 0.83, 0.93) (Table 2).

**Table 2:** Associations of six systemic inflammatory indices and ischemic stroke, using logistic regression

Characteristic^a^	Discovery dataset (SAH-SFMU)	Validation dataset (DPH)
	Odds ratio (95% CI)	Odds ratio (95% CI)
Model 1		
SIRI	8.73 (7.57, 10.10)^b^	1.00 (0.97, 1.03)
SII	1.03 (1.01, 1.04)^b^	1.00 (0.99, 1.01)
PIV	1.01 (1.01, 1.01)^b^	0.99 (0.99, 1.01)
NLR	2.30 (2.17, 2.45)^b^	1.05 (1.01, 1.10)^b^
PLR	1.01 (1.01, 1.01)^b^	0.99 (0.99, 1.01)
LMR	0.76 (0.74, 0.77)^b^	0.85 (0.80, 0.89)^b^
Model 2		
SIRI	7.29 (6.32, 8.45)^b^	1.00 (0.96, 1.02)
SII	1.03 (1.01, 1.04)^b^	1.00 (0.99, 1.01)
PIV	1.01 (1.01, 1.01)^b^	0.99 (0.99, 1.00)
NLR	2.20 (2.06, 2.34)^b^	1.05 (1.01, 1.10)^b^
PLR	1.01 (1.01, 1.01)^b^	1.00 (0.99, 1.00)
LMR	0.78 (0.76, 0.79)^b^	0.86 (0.82, 0.91)^b^
Model 3		
SIRI	8.77 (7.48, 10.33)^b^	0.99 (0.95, 1.02)
SII	1.03 (1.01, 1.04)^b^	1.00 (0.99, 1.01)
PIV	1.01 (1.01, 1.01)^b^	0.99 (0.99, 1.01)
NLR	2.23 (2.08, 2.39)^b^	1.04 (0.99, 1.09)
PLR	1.01 (1.01, 1.01)^b^	0.99 (0.99, 1.00)
LMR	0.77 (0.75, 0.78)^b^	0.88 (0.83, 0.93)^b^

^a^*Model 1* was unadjusted. *Model 2* adjusted glucose and blood lipids profile (total cholesterol, triglycerides, low-density lipoprotein cholesterol, high-density lipoprotein cholesterol). *Model 3* further adjusted hemoglobin, uric acid, total protein, and calculated globulin based on Model 2

^b^Statistically significant at α = .05

*IS* ischemic stroke, *SAH-SFMU* the Second Affiliated Hospital of Shandong First Medical University, *DPH* Dongping People’s Hospital, *CI* confidence interval, *SIRI* systemic inflammation response index, *SII* systemic immune-inflammation index, *PIV* pan-immune-inflammation value, *NLR* neutrophil lymphocyte ratio, *PLR* platelet lymphocyte ratio, *LMR* lymphocyte monocyte ratio

### Classification models

**Fig. 3:**
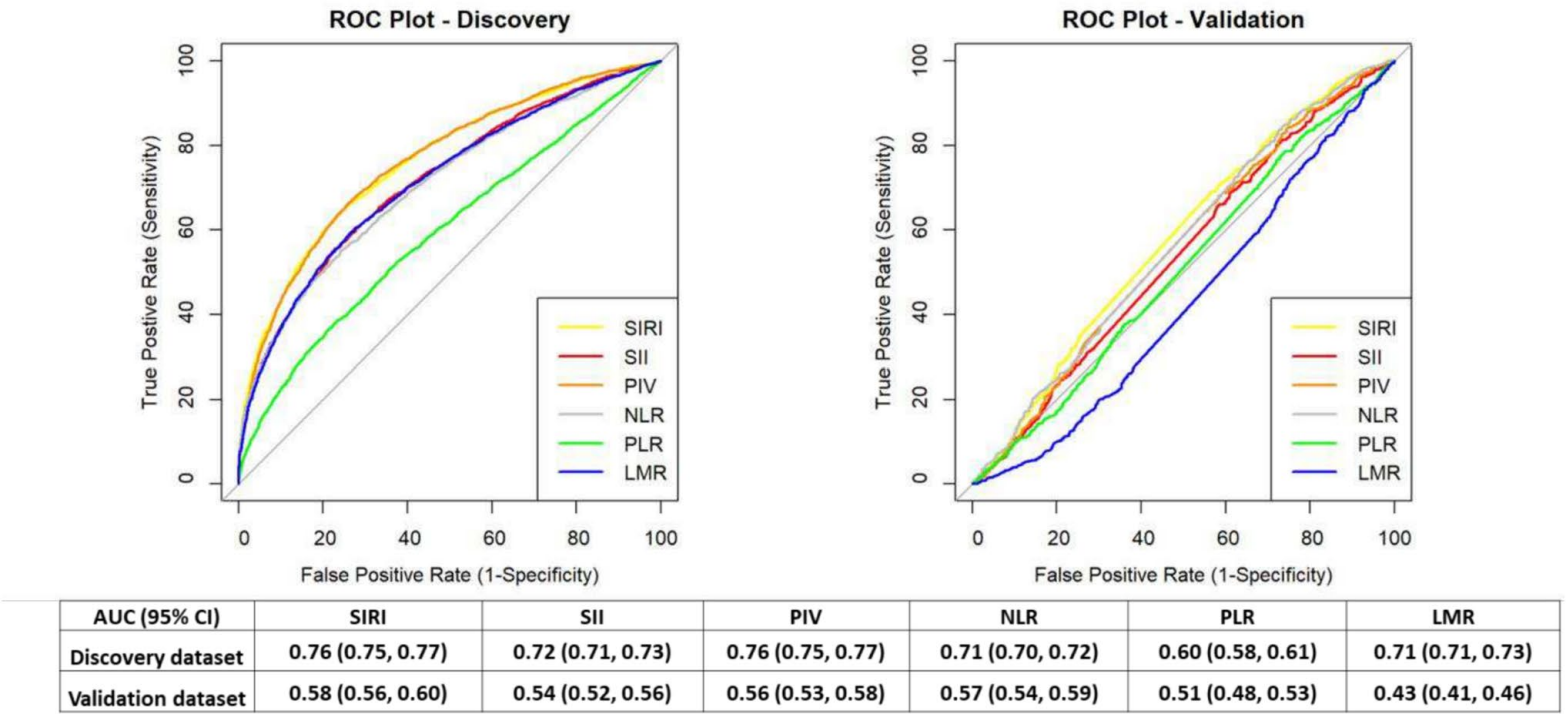
Comparison of the ROC curves generated by six systemic inflammatory indices. *ROC* receiver operating characteristic, *AUC* area under the curve, *SIRI* systemic inflammation response index, *SII* systemic immune-inflammation index, *PIV* pan-immune-inflammation value, *NLR* neutrophil lymphocyte ratio, *PLR* platelet lymphocyte ratio, *LMR* lymphocyte monocyte ratio

Single systemic inflammatory index demonstrated accurate classification of IS-present cases from healthy controls in the discovery dataset, with the AUC values ranging from 0.60 to 0.76. Notably, SIRI exhibited the highest performance for discriminating IS-present cases and healthy controls (AUC 0.76, 95% CI 0.75, 0.77). In the validation dataset, however, these indices were unable to accurately distinguish the IS-present cases from IS-absent controls, with AUCs ranging from 0.43 to 0.58 (Fig. 3).

### Sensitivity analyses

Sensitivity analyses confirmed consistent association patterns when stratified by age and sex. In the discovery dataset, all systemic inflammatory indices were associated with IS across age and sex. Conversely, in the validation dataset, none of six indices was associated with IS in women. LMR showed associations with IS among middle-aged adults, older adults, and men in the validation set, with ORs (95% CIs) of 0.89 (0.81, 0.96), 0.88 (0.82, 0.94), and 0.79 (0.73, 0.86), respectively. Additional information is provided in the Supplementary Material.

## Discussions

In this study, we investigated associations between six systemic inflammatory indices and ischemic stroke (IS) within a middle-aged and older Chinese population. The results underscore the significance of these indices, which integrate platelets with various white blood cell subsets, in classifying IS risk. After adjusting for potential confounding factors, IS was found to be associated with all six systemic indices in the discovery dataset. Notably, the significant association between lymphocyte monocyte ratio (LMR) and ischemic stroke remained significant in the validation dataset when comparing IS patients with IS-absent patients having IS mimics.

The immune response to acute cerebral ischemia plays a pivotal role in stroke pathogenesis, regardless of stroke type [5]. Atherosclerosis, a major contributor to ischemic strokes, is inherently an inflammatory disease [24]. In addition to classical risk factors, maladaptive immune mechanisms can further heighten the risk of stroke [25]. Severe systemic inflammation post-ischemic stroke may exacerbate the process of injury in stroke [26]. One important predictive inflammatory response indicator is the measured change in peripheral blood cell composition [27]. Since the goal of treatment for acute ischemic stroke is fundamentally to save as many ischemic areas at risk of infarction as possible to save the brain and improve functional outcomes [28]. It is important to continue to prevent or treat complications such as pneumonia and other infections, thrombosis or seizures, as well as good rehabilitation and prognostic care, which not only reduces neurological dysfunction but also helps patients return to daily function, while preventing and treating long-term sequelae such as spasms, post-stroke depression or post-stroke dementia [29]. Identifying sensitive and specific inflammatory biomarkers for stroke patients enables imperative for personalized treatment and advancements in precision medicine.

LMR, the ratio of lymphocyte count and monocyte count, was the only index that significantly associated with IS in both discovery (IS-present cases *vs* healthy controls; OR 95% CI is 0.77 [0.75–0.78]) and validation (IS-present cases *vs* IS mimics; OR 95% CI is 0.88 [0.83–0.93]) datasets. Our finding is consistent with previous findings in several clinical studies [30–32], in those research LMR is inversely associated with stroke severity or a reduction in lymphocytes contributes to a poor outcome in patients with acute IS. Potential mechanism may be because that the reduction in lymphocyte counts occurs through a cascade where stroke-related stress triggers the renin-angiotensin system, elevates cortisol levels, and promotes lymphocyte apoptosis [32]. Moreover, the proportion of monocytes was observed increased in patients with IS compared with controls without IS [19]. Prior research suggested that monocytes disrupt the blood–brain–barrier as elevated numbers of circulating monocytes are linked to the hyperintense acute reperfusion marker, a larger acute IS volume and poorer outcomes [33]. LMR's superior performance compared to other inflammatory indices might be attributed to its ability to capture both the immunosuppressive state (lymphopenia) and inflammatory response (monocytosis) simultaneously, making it a more comprehensive marker of the immune response in IS.

By examining electronic medical records from two independent hospitals in Shandong Province, China, we provide robust evidence for the associations between systemic inflammatory indices and IS. In the current study, the observed positive associations between systemic inflammatory indices (SIRI, SII, PIV, NLR, PLR) and IS in comparison to healthy controls align with previous research linking inflammation to cerebrovascular events or related risk factors, such as sleep-related disorder [34, 35]. These indices, reflective of the intricate interplay between neutrophils, monocytes, lymphocytes, and platelets, could serve as potential biomarkers for identifying individuals at heightened risk of IS. Notably, the significant elevation in the odds of IS with increasing SIRI, even after adjustments for glucose, lipids, and other relevant factors, highlights the robust association with IS. It is remarkable that controls in validation dataset IS-absent which means these individuals with IS mimics, such as other chronic inflammatory diseases. It also hints that these systemic inflammatory indices (SIRI, SII, PIV, NLR, PLR) are generalized for inflammation but not specific for IS that may explain the reason for the association between systemic inflammatory indices and IS was not validated in the validation dataset.

Despite these positives, our study has certain limitations. The case–control study design makes it difficult to determine causation or directionality of the link between systemic inflammatory indices and ischemic stroke. Furthermore, possible underestimate may occur since individuals from poor socioeconomic backgrounds (tended to with unhealthy body weight and poor lifestyle) were underrepresented. EMR typically lacks comprehensive documentation of socioeconomic status, lifestyle factors, and related health metrics, which could impact our findings. To further understand the pathophysiology of inflammation in ischemic stroke, future research should use longitudinal designs [36, 37], take into account of gene-tissue-environment interactions [38–40], and conduct casual effect studies [41, 42]. This research might help elucidate the causal relationship between systemic chronic inflammation and ischemic strokes. Understanding these mechanisms could ultimately help reduce the healthcare burden at the community level [43].

## Conclusions

In summary, this study bridges existing knowledge gaps by exploring and validating associations between specific systemic inflammatory indices and IS. In doing so, it contributes to the evolving landscape of cerebrovascular research, emphasizing the importance of inflammation in the context of ischemic stroke and laying the foundation for potential advancements in risk prediction and therapeutic strategies. While those inflammatory indices are potential indicators for distinguishing IS from healthy conditions, additional biomarkers may be needed when differentiating IS from other chronic inflammatory conditions in clinical practice.

## Supplementary Information

Below is the link to the electronic supplementary material.

Supplementary file1 (DOCX 30 KB)

## Data Availability

The data are available from the corresponding authors on a reasonable request.
